# Quality Evaluation of Scrophulariae Radix Processed by Different ‘Sweating’ Methods Based on Simultaneous Determination of Multiple Bioactive Constituents Combined with Grey Relational Analysis

**DOI:** 10.3390/molecules21070850

**Published:** 2016-06-28

**Authors:** Shengnan Wang, Yujiao Hua, Li Xu, Lisi Zou, Xunhong Liu, Yiyuan Luo, Juanxiu Liu, Ying Yan

**Affiliations:** 1 College of Pharmacy, Nanjing University of Chinese Medicine, Nanjing 210023, China; jshmwsn@163.com (S.W.); huayujiao1020@163.com (Y.H.); ggn0117@sohu.com (L.Z.); luoyiyuan0012@sohu.com (Y.L.); liujx0516@163.com (J.L.); yanying93happy@sina.com (Y.Y.); 2 Yangzhou Institute for Drug Control, Yangzhou 225100, China; Babyjs1225@126.com

**Keywords:** Scrophulariae Radix, ‘sweating’, UPLC-QTRAP-MS/MS, simultaneous determination, grey relational analysis

## Abstract

Scrophulariae Radix is one of the most popular traditional Chinese medicines (TCMs), which needs to be processed by ‘sweating’ methods. Primary processing of Scrophulariae Radix is an important link which closely relates to the quality of products in this TCM. To facilitate selection of the suitable ‘sweating’ processing method for Scrophulariae Radix, in this study the quality of Scrophulariae Radix processed by different ‘sweating’ methods was evaluated based on simultaneous determination of multiple bioactive constituents combined with grey relational analysis. The contents of iridoid glycosides, phenylpropanoid glycosides, and organic acids in Scrophulariae Radix processed by different ‘sweating’ methods were simultaneously determined using ultra high performance liquid chromatography coupled with triple quadrupole-linear ion trap mass spectrometry (UPLC-QTRAP-MS/MS). Furthermore, grey relational analysis (GRA) was performed to evaluate the ‘sweating’ processed samples according to the contents of twelve constituents. All of the results demonstrated that the quality of Scrophulariae Radix processed by oven drying at 35 °C and ‘sweating’ for three days was better. The developed method was useful for the overall assessment on quality of Scrophulariae Radix, and this study may provide the foundation and support for ‘sweating’ processing of Scrophulariae Radix in normalization and standardization.

## 1. Introduction

Scrophulariae Radix (Xuanshen in Chinese), derived from the dried root of *Scrophularia ningpoensis* Hemsl., is one of the most popular traditional Chinese medicines (TCMs), and is officially documented in the Chinese Pharmacopoeia. It has been used for thousands of years in China for its excellent traditional therapeutic effects on blood cooling, yin nourishing, fire purging, and toxin removal [1]. Modern pharmacological studies and clinical practice demonstrated that it possesses anti-angiogenesis [2], ventricular remodeling [3,4], anti-inflammatory [5], and antimicrobial [6] activities, and can be used for the treatment of rheumatism, pharyngalgia, arthritis, constipation, and conjunctival congestion [7]. Phytochemical investigation has revealed that Scrophulariae Radix mainly contains several types of constituents, such as iridoid glycosides, phenylpropanoid glycosides, and organic acids [8,9,10,11]. A recent research proved that these constituents contained in Scrophulariae Radix could exhibit various biological activities, including inhibiting macrophage functions involved in the inflammatory process and antioxidative activity for reducing the oxidized OH adducts of dAMP and dGMP [12,13,14,15], enhancing immune activity [16], protecting the liver [17], and decreasing levels of hyaluronic acid [18]. The synergistic action of these various components is considered to be responsible for the therapeutic effects of Scrophulariae Radix.

A variety of analytical methods, including high-performance liquid chromatography [19,20,21,22], capillary electrophoresis [23], and ultra-performance liquid chromatography [24,25] were established for analyzing chemical constituents of Scrophulariae Radix, but these methods only focused on the determination of iridoid glycosides and/or phenylpropanoid glycosides. There is no method for simultaneous determination of iridoid glycosides, phenylpropanoid glycosides, and organic acids. It is well known that the content of a few index compounds might not accurately reflect the quality of the complex herbal products. Therefore, it was necessary to develop a rapid and sensitive method to simultaneously determine iridoid glycosides, phenylpropanoid glycosides, and organic acids for the quality control of Scrophulariae Radix. The developed ultra high performance liquid chromatography coupled with triple quadrupole-linear ion trap mass spectrometry (UPLC-QTRAP-MS/MS) method is highly sensitive, strongly selectivity, quantitatively accurate, and results in fast analysis. Compared with the HPLC method, the developed method has the advantages of good separation efficiency, high peak capacity, and high sensitivity, which is more suitable for the separation and analysis of the complex systems of traditional Chinese medicines (TCMs). UPLC-QTRAP-MS/MS uses multiple reaction monitoring (MRM) for quantitative analysis, and the neutral fragment scan is used for the target material. It can solve the problem that the UPLC method is difficult to separate the complex systems. Not only does the quadrupole linear ion trap system retain all of the traditional functions of the standard triple quadrupole but, also in the linear ion trap (LIT) mode, the QTRAP system can improve the sensitivity of the full scan mode, enhance the ion scan (EPI), and provide the multipole ion scan (MS^3^) functions [26,27,28].

Primary processing of Scrophulariae Radix is an important link which closely relates to the quality of products in this TCM. According to the Chinese Pharmacopoeia (2015), Scrophulariae Radix needs to be processed by ‘sweating’ methods. ‘Sweating’ is a traditional technology for primary processing of Chinese herbal medicine, which was derived from long-term practices and experiences. A Chinese herbal medicine was baked with a micro fire to semi-dry, or micro cooked and micro steamed, then was piled up to sweat to make the internal moisture spillover during the procedure of processing. The ‘sweating’ process makes the Chinese herbal medicine become soft and change color to increase fragrance or reduce irritation, which is conducive to drying. However, due to the difference of ‘sweating’ methods during primary processing, the variation of effective components contained in this TCM is large, and its quality is uneven. The key to the manufacturing procedure of Scrophulariae Radix is how to ensure its high and uniform quality.

The aim of this paper was to evaluate the quality of Scrophulariae Radix processed by different ‘sweating’ methods based on simultaneous determination of iridoid glycosides (harpagide, harpagoside, catalpol, and aucubin), phenylpropanoid glycosides (angoroside C, verbascoside), and organic acids (fumaric acid, caffeic acid, *p*-coumaric acid, ferulic acid, cinnamic acid, and *p*-methoxycinnamic acid) in this TCM. Combined with grey relational analysis. UPLC-QTRAP-MS/MS was successfully applied to analyze twelve samples processed by different ‘sweating’ methods. Furthermore, grey relational analysis (GRA) was performed to evaluate the ‘sweating’ processed samples according to the contents of twelve constituents.

## 2. Results and Discussion

### 2.1. Optimization of Sample Preparation

In order to achieve an efficient extraction of bioactive constituents in Scrophulariae Radix, key factors, such as methanol concentration (50%, 60%, 70%, 80%, 90%, and 100%, *v*/*v*), sample-solvent ratio (1:20, 1:40, 1:60, and 1:80, *v*/*v*), and ultrasonic time (15, 30, 45, 60, and 75 min) were investigated by single variable investigation. The optimum sample extraction condition was achieved by using of 70% (*v*/*v*) methanol, 20 times, for 60 min. All of the samples were extracted at room temperature.

### 2.2. Optimization of UPLC-QTRAP-MS/MS Conditions

**Figure 1:**
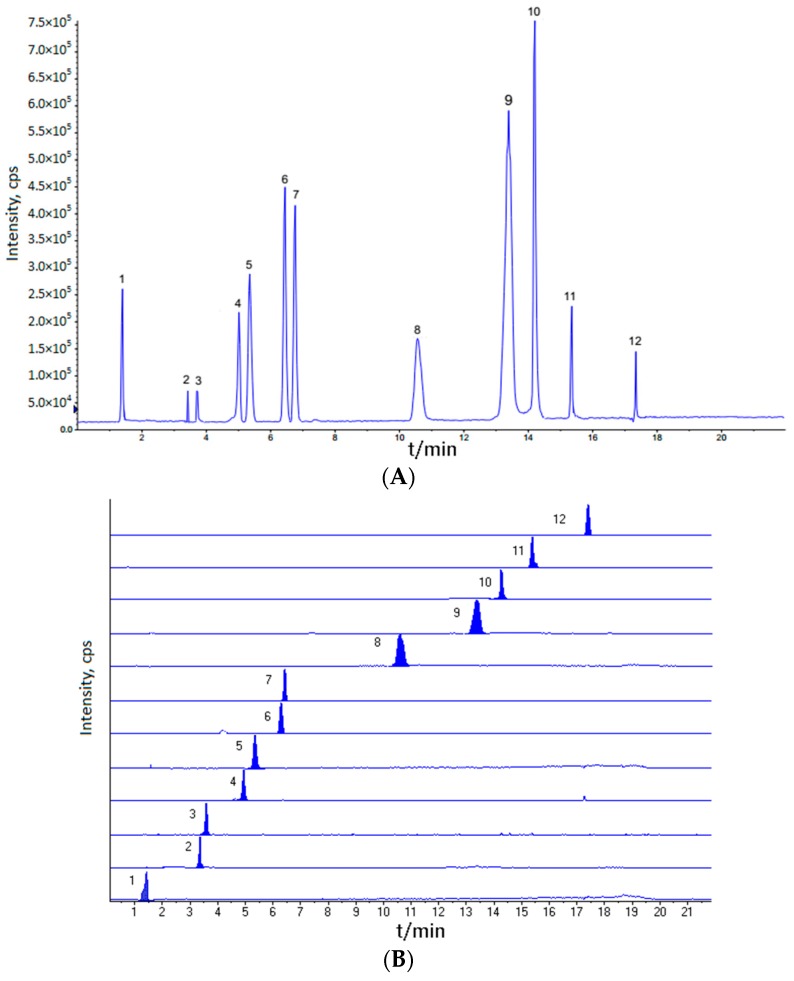
Total ion chromatograms (TIC) (**A**); and multi-reaction monitoring (MRM) (**B**), of the 12 analytes.

To obtain the optimum elution conditions, various UPLC parameters including a mobile phase modifier (methanol-water, acetonitrile-water), column temperature (35, 40 and 45 °C), and flow rate (0.8, 1.0, and 1.2 mL/min). Based on sensitivity, the optimum UPLC conditions were obtained when the mobile phase modifier was acetonitrile-water, column temperature was 35 °C, and flow rate was 1.0 mL/min. Preliminary experiments were conducted with the purpose of finding the best instrumental conditions. Total ion chromatograms (TIC) and multi-reaction monitoring (MRM) of the 12 analytes are presented in Figure 1. MS conditions were studied in both positive and negative modes. After trial and error inspection, we found that iridoid glycosides, phenylpropanoid glycosides, and organic acids have a good condition in the negative ion mode. We summarize the optimum values for each condition of twelve analytes (Table 1). The results showed that the highest sensitivity was obtained at a certain value of fragmentor or collision energy (CE). For example, fumaric acid had the highest sensitivity at fragmentor-55 and CE-11.

### 2.3. Method Validation

#### 2.3.1. Calibration Curves, Limit of Detection (LOD), and Limit of Quantitation (LOQ)

The calibration curve for each analyte was obtained in duplicate with at least six appropriate concentrations. The correlation coefficients of all target components exceeded 0.9988 with good linearity. The LOD and LOQ of 12 analytes were measured at signal-to-noise ratios of 3 and 10, and the ranges were 1.29–9.45 and 2.07–19.65 ng/mL, respectively. The results are reported in Table 2.

#### 2.3.2. Precision, Stability, and Recovery

Intra- and inter-day variations were used to evaluate method precision. For the intra-day variability test, the mixed standard solutions were analyzed for six replicates within a day; for the inter-day variability test, the solutions were examined for three consecutive days. The relative standard deviation (RSD) was taken as a measure of precision. The RSD values of intra- and inter-day variations of the 12 analytes were in the range of 0.96%–4.35% and 1.65%–6.87%, respectively. The results are shown in Table 3.

A stability test was further performed to analyze the variations in the sample solutions at 0, 2, 4, 8, 12, and 24 h, respectively. The stability present as percentage of the original value was more than 89.56%. The results are shown in Table 4.

A recovery test was used to evaluate the accuracy of this method. The test was performed by adding the corresponding marker compounds at low (80% of the known amounts), medium (same as the known amounts), and high (120% of the known amounts) levels to the Scrophulariae Radix sample which had previously been analyzed. The mixture was extracted and analyzed using the aforementioned method in triplicate. The overall recoveries laid between 93.50% and 97.60% with RSDs between 1.96% and 3.60%. The results are shown in Table 3.

### 2.4. Quantitative Analysis of Sample

The developed UPLC-QTRAP-MS/MS method was subsequently applied to the comprehensive quality evaluation of 12 batches of Scrophulariae Radix samples processed by different ‘sweating’ methods. The results of the quantitative determination of the 12 analytes from these samples are summarized in Table 5. The results indicated that there were significant differences in total contents of iridoid glycosides, phenylpropanoid glycosides, and organic acids among twelve samples. Iridoid glycosides (mainly harpagide) were the highest and ranged from 0.1406 to 6.2461 mg/g. Phenylpropanoid glycosides (mainly angoroside C) were the second highest in the ranges of 8.5551–8.8068 mg/g. Organic acids (mainly fumaric acid) were the least ranging from 0.0303 to 7.2058 mg/g. Due to the differences of twelve bioactive constituents in Scrophulariae Radix processed by different ‘sweating’ methods, it was difficult to intuitively evaluate the quality of this TCM.

### 2.5. GRA of the Sample

To further evaluate the variation of iridoid glycosides, phenylpropanoid glycosides, and organic acids in all tested samples, GRA (grey relational analysis) was performed on the basis of the contents of 12 target compounds. It could be seen that the quality of the sample (S5) processed by oven drying at 35 °C and ‘sweating’ for three days was better according to the results shown in Table 6.

## 3. Materials and Methods

### 3.1. Chemicals and Reagents

**Figure 2:**
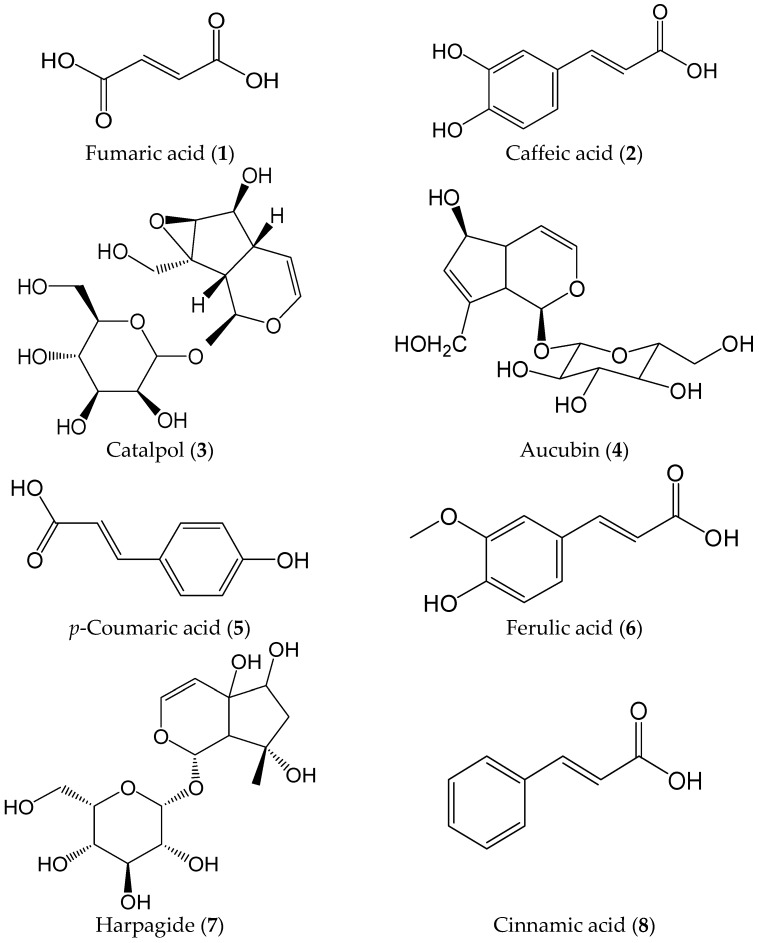

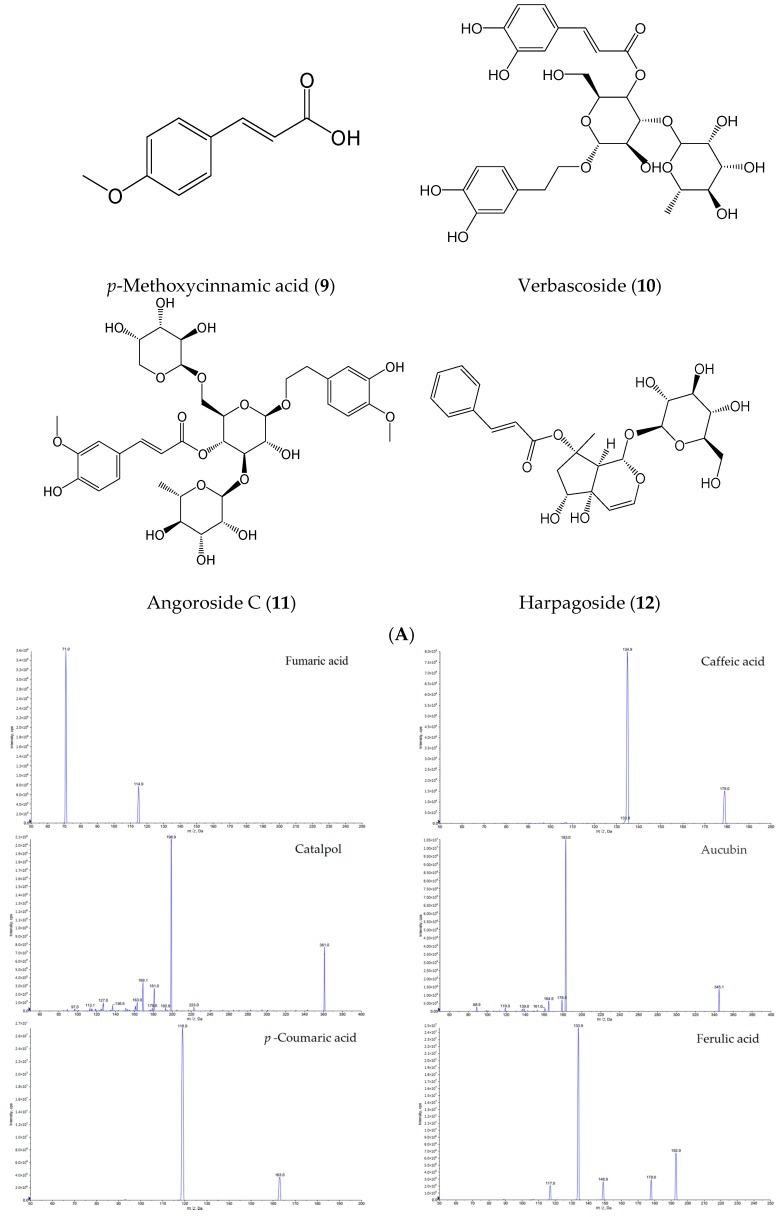

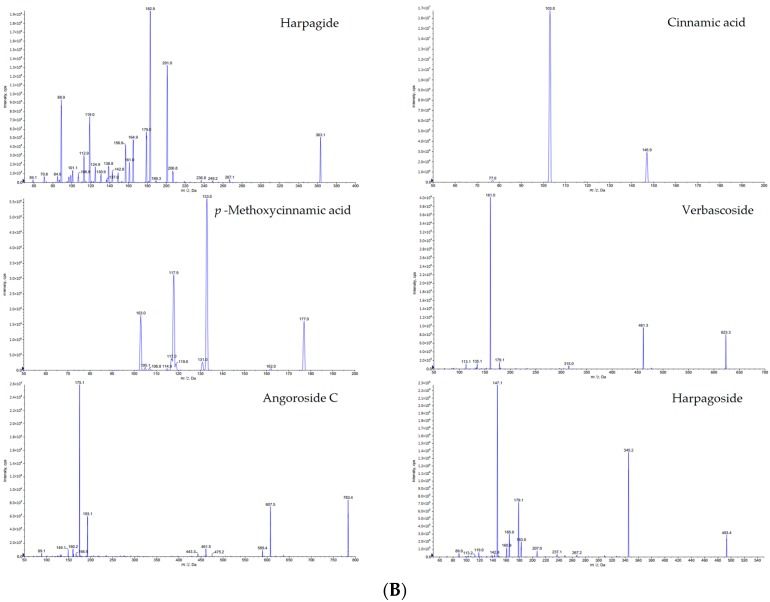
Chemical structures of the twelve compounds analyzed in the study (**A**); and mass spectrums of the twelve compounds in negative mode (**B**).

The chemical standards harpagide (**1**), harpagoside (**2**), ferulic acid (**3**), catalpol (**4**), aucubin (**5**), cinnamic acid (**6**) , and caffeic acid (**7**) were purchased from the Chinese National Institute of Control of Pharmaceutical and Biologiceal Products (Beijing, China); angoroside C (**8**) was purchased from Chengdu Chroma-Biotechnology Co., Ltd. (Chengdu, China); verbascoside (**9**) was purchased from Shanghai Yongye-Biotechnology Co., Ltd. (Shanghai, China); *p*-Coumaric acid (**10**) and *p*-methoxycinnamic acid (**11**) were purchased from Shanghai Yuanye-Biotechnology Co., Ltd. (Shanghai, China); fumaric acid (**12**) was purchased from Sinopharm chemical reagent Co., Ltd. (Nanjing, China). The purity of all chemical standards is greater than 99% (Figure 2). Methanol and acetonitrile of HPLC grade were purchased from Merck (Damstadt, Germany). Ultrapure water was prepared using a Milli-Q purifying system (Millipore, Bedford, MA, USA) under a resistivity of 18.2 MΩ/cm, other reagent solutions were analytical grade (Sinopharm Chemical Reagent Co., Ltd., Shanghai, China).

### 3.2. Plant Materials

The roots of *Scrophularia ningpoensis* were collected from Jinyun in Zhejiang Province, China in November 2014. The botanical origin of the materials was identified by Professor Xunhong Liu (Department for Authentication of Chinese Medicines, Nanjing University of Chinese Medicine, China), and the voucher specimens were deposited at the Herbarium in Nanjing University of Chinese Medicine, China. The procedure of ‘sweating’ is as follows: remove the rhizome, germ, fibrous root, and sediment from the fresh herb, dry to semi-dry the product, put the Scrophulariae Radix into the black bag for several days as the first ‘sweating’; then take out the Scrophulariae Radix from the black bag, dry to semi-dry the product, put the Scrophulariae Radix into the black bag for several days as the second ‘sweating’; then take out the Scrophulariae Radix from the black bag, dry to semi-dry the product, put the Scrophulariae Radix into the black bag for several days as the third ‘sweating’; then take out the Scrophulariae Radix from the black bag, dry to totally dry the final product. The detailed information of twelve samples processed by different ‘sweating’ methods was summarized in Table 7.

### 3.3. Preparation of Standard Solutions

A mixed standard stock solution containing the twelve reference compounds **1**–**12** was prepared by dissolving them in methanol and their concentrations were as follows: **1**, 709.00 μg/mL; **2**, 22.25 μg/mL; **3**, 175.50 μg/mL; **4**, 914.00 μg/mL; **5**, 53.00 μg/mL; **6**, 128.00 μg/mL; **7**, 569.00 μg/mL; **8**, 126.80 μg/mL; **9**, 426.00 μg/mL; **10**, 1744.00 μg/mL; **11**, 470.00 μg/mL; and **12**, 198.00 μg/mL. This solution was then diluted with methanol to a series of appropriate concentrations for construction of calibration curves. All of the solutions were stored at 4 °C and were filtered through a 0.22 μm membrane (Jinteng laboratory equipment Co., Ltd., Tianjing, China) prior to injection.

### 3.4. Preparation of Sample Solutions

One gram of Scrophulariae Radix powder, after passing through a 40 mesh sieve, was weighed accurately and ultrasonically extracted with 20 mL 70% (*v*/*v*) methanol for 60 min. The extract solution was cooled at room temperature and subsequently centrifuged at 12,000 rpm for 10 min. All of the solutions were stored at 4 °C and filtered through a 0.22 μm membrane prior to injection.

### 3.5. Chromatographic and Mass Spectrometric Conditions

UPLC was performed by using a Shimadzu UPLC-20ADXR system (Shimadzu, Kyoto, Japan). All separations were carried out on a BDS HYPERSIL C_18_ (250 mm × 4.6 mm i.d., 5 μm). The mobile phase consisted of A (water) and B (acetonitrile) with a gradient elution: 0–1 min, 5% B; 1–10 min, 5%–20% B; 10–14 min, 20%–35% B; 14–16 min, 35%–75% B; 16–18.1 min, 75%–5% B; and the re-equilibration time was 4 min. The flow rate of the mobile phase was 1.0 mL/min, and the column temperature was maintained at 35 °C. The injection volume was 2 μL.

Mass spectrometry was performed on an API 4000 triple quadrupole mass spectrometer (AB SCIEX, Framingham, MA, USA) equipped with an electrospray ionization (ESI) source operating in the negative ion mode. The parameters in the source were set as follows: GS1 flow 65 L/min, GS2 flow 65 L/min, CUR flow 30 L/min; gas temperature 650 °C; pressure of nebulizer of MS −4500 V. The analyte detection was performed by using multiple reaction monitoring (MRM).

### 3.6. Validation of the Method

A series of analyses such as the linearity, stability, precision, and LOD and LOQ were conducted to validate the performance of the method. The standard solution containing 12 markers was prepared and diluted with methanol to appropriate concentrations for the construction of calibration curves. Calibration curves were developed by plotting the peak areas versus the corresponding concentrations of each analyte. The precision of the method was evaluated by analyzing the 12 standard compounds. The RSD of the peak area was used to evaluate the precision of the developed method. The LOD and LOQ of 12 analytes were measured at signal-to-noise ratios of 3 and 10, respectively. The recovery test was performed by adding the corresponding index compounds with high (120%), middle (100%), and low (80%) levels into accurately-weighed samples. The spiked samples were then extracted and analyzed using the aforementioned method, and triplicate experiments were performed at each level.

### 3.7. Grey Relational Analysis (GRA)

In order to evaluate quality of Scrophulariae Radix processed by different ‘sweating’ methods, GRA was carried out by the following steps.

#### 3.7.1. The Establishment of Sample Dataset

Determination of the contents of twelve bioactive constituents in Scrophulariae Radix processed by different ‘sweating’ methods was conducted. A grey pattern recognition dataset of the quality of Scrophulariae Radix according to the contents of twelve bioactive constituents was established.

#### 3.7.2. Normalization Treatment of Raw Data

Assume that there were *n* samples; each sample had *m* indexes. Compose the evaluation unit sequence as {*X*_ik_}, (i = 1, 2, 3,…*n*; k = 1,2,3,…*m*; *n* = 12, *m* = 12 in this experiment). Normalize the raw data according to the formula due to the difference of unified measuring unit.

*Y*_ik_ = *X*_ik_/*X*_k_ (1)

Note: *Y*_ik_ is the data after normalization treatment, *X*_ik_ is the raw data, *X*_k_ is the mean value of the k-th index of the *n*-th sample.

#### 3.7.3. The Establishment of the Optimal Reference Sequence and the Worst Reference Sequence

With grey relational analysis, we should choose the reference sequence. Set the optimum reference sequence and worst reference sequence as {*X*_sk_} and {*X*_tk_}, respectively (k = 1, 2, 3,... *m*). Each index of the optimum reference sequence is the maximum value of the corresponding index of the *n*-th sample, namely {*X*_sk_}; each index of the worst reference sequence is the minimum value of the corresponding index of the *n*-th sample, namely {*X*_tk_}. Calculate the D-value of each assessment unit relative to the optimal (poor) reference sequence.

For the optimal reference sequence, the correlation coefficient is:

(2) $$\begin{array}{l} \xi_{k\left(s\right)}^{i}=\frac{\Delta_{\text{min}}\;+\;{\rho \Delta }_{\text{max}}}{\left|Y_{\text{ik}}-Y_{\text{sk}}\right|\;+\;{\rho \Delta }_{\text{max}}}\;(\Delta_{\text{min}}\;=\;\min|Y_{\text{ik}}\;-\;Y_{\text{sk}}|,\;\Delta_{\text{max}}\;=\;\max|Y_{\text{ik}}\;-\;Y_{\text{sk}}|,\\ i=1,2,3,\ldots n;\;k\;=\;1,2,3,\ldots m) \end{array}$$

For the worst reference sequence, the correlation coefficient is:

(3) $$\begin{array}{l} \xi_{k\left(t\right)}^{i}=\frac{\Delta_{\text{min}}^{\prime }+{\rho \Delta }_{\text{max}}^{\prime }}{\left|Y_{\text{ik}}-Y_{\text{tk}}\right|+{\rho \Delta }_{\text{max}}^{\prime }}\;(\Delta_{\text{min}}^{\prime }\;=\;\min|Y_{\text{ik}}\;-\;Y_{\text{tk}}|,\;\Delta_{\text{max}}^{\prime }\;=\;\max|Y_{\text{ik}}\;-\;Y_{\text{tk}}|,\\ i=1,\;2,\;3,\;\ldots \;n;\;k\;=\;1,\;2,\;3,\;\ldots \;m;\;\rho :\;\text{identification coefficient},\;\rho \;=\;0.5) \end{array}$$

#### 3.7.4. Calculation of Correlation Coefficient and Correlative Degree

Calculate the correlation coefficient and correlative degree of each assessment unit relative to optimal (poor) reference sequence:

(4) $$r_{i\left(s\right)}=\frac{1}{m}\overset{m}{\underset{k=\;1}{\sum }}\xi_{k\left(s\right)}^{i}$$

(5) $$r_{i\left(t\right)}=\frac{1}{m}\overset{m}{\underset{k=1}{\sum }}\xi_{k\left(t\right)}^{i}$$

#### 3.7.5. Calculation of Relative Correlative Degree

Calculate the relative correlative degree of each sample:

(6) $$r_{i}=\frac{r_{i\left(s\right)}}{r_{i\left(s\right)}+r_{i\left(t\right)}}$$

## 4. Conclusions

In this study, an efficient and sensitive UPLC-QTRAP-MS/MS method has been developed and validated for the simultaneous determination of iridoid glycosides, phenylpropanoid glycosides, and organic acids in Scrophulariae Radix. The validated method was successfully applied to quantify twelve bioactive constituents in Scrophulariae Radix processed by different ‘sweating’ methods. Furthermore, grey relational analysis (GRA) was performed to evaluate the ‘sweating’ processed samples according to the contents of twelve constituents. All of the results demonstrated that the quality of Scrophulariae Radix processed by oven drying at 35 °C and ‘sweating’ for three days was better. The developed method was useful for the overall assessment on quality of Scrophulariae Radix, and this study may provide the foundation and support for ‘sweating’ processing of Scrophulariae Radix in normalization and standardization.

## Figures and Tables

**Table 1:** Precursor/product ion pairs and parameters for MRM of compounds used in this study.

No.	Compound	*t_R_* (min)	[M − H]^−^ (*m/z*)	MRM Transitions (Precursor→Product)	Fragmentor (V)	Collision Energy (eV)
1	Fumaric acid	1.42	114.9	114.9→71.0	−55	−11
2	Caffeic acid	3.34	179.0	179.0→134.9	−52	−23
3	Catalpol	3.59	361.0	361.0→198.9	−76	−11
4	Aucubin	5.02	345.1	345.1→183.1	−65	−13
5	*p*-Coumaric acid	5.35	163.0	163.0→118.9	−56	−19
6	Ferulic acid	6.38	192.9	192.9→133.9	−54	−24
7	Harpagide	6.42	363.1	363.→182.9	−90	−21
8	Cinnamic acid	10.57	146.9	146.9→103.0	−64	−16
9	*p*-Methoxycinnamic acid	13.38	177.0	177.0→133.0	−52	−16
10	Verbascoside	14.19	623.3	623.3→161.0	−116	−57
11	Angoroside C	15.34	783.4	783.4→175.1	−129	−55
12	Harpagoside	17.34	493.4	493.4→147.1	−65	−31

**Table 2:** Regression equation, correlation coefficients, linearity ranges, and limits of detection (LOD) and quantitation (LOQ) of the 12 investigated compounds.

No.	Compound	Calibration Curves	*r^2^*	Linear Range (μg/mL)	LOD (ng/mL)	LOQ (ng/mL)
1	Fumaric acid	y = 254.90x – 708.31	0.9996	0.709–709.000	9.13	18.89
2	Caffeic acid	y = 1149.30x + 1096.9	0.9994	0.011–22.250	1.29	2.15
3	Catalpol	y = 27.61x – 84.26	0.9998	0.088–175.500	3.10	8.69
4	Aucubin	y = 128.38x + 131.04	0.9994	0.457–914.000	5.62	12.33
5	*p*-Coumaric acid	y = 3854.90x – 2322.20	0.9998	0.026–53.000	1.88	2.07
6	Ferulic acid	y = 950.10x – 1032.90	0.9988	0.064–128.000	3.47	9.56
7	Harpagide	y = 81.58x + 275.52	0.9996	0.284–569.000	3.94	10.75
8	Cinnamic acid	y = 1809.60x + 3360.20	0.9988	0.063–126.800	4.11	8.42
9	*p*-Methoxycinnamic acid	y = 1776.50x + 5756.80	0.9996	0.213–426.000	4.21	9.68
10	Verbascoside	y = 203.29x +1645.80	0.9996	0.872–1744.000	9.45	19.65
11	Angoroside C	y = 178.95x +1045.10	0.9990	0.253–470.000	3.85	8.71
12	Harpagoside	y = 115.78x + 596.85	0.9996	0.099–198.000	3.47	7.25

**Table 3:** Precision and recovery of the 12 investigated compounds.

No.	Compound	Precision (RSD, %)		Recovery (%, *n* = 3)	
		Intraday (*n* = 6)	Interday (*n* = 6)	Mean	RSD (%)
1	Fumaric acid	2.59	4.86	97.60	1.96
2	Caffeic acid	4.35	3.73	95.64	2.29
3	Catalpol	2.55	2.38	97.18	2.66
4	Aucubin	3.81	4.76	95.16	2.38
5	*p*-Coumaric acid	4.08	2.70	95.66	3.43
6	Ferulic acid	3.81	6.87	95.39	2.97
7	Harpagide	1.68	1.65	94.98	2.26
8	Cinnamic acid	2.50	2.37	95.73	2.76
9	*p*-Methoxycinnamic acid	2.81	1.69	95.85	2.08
10	Verbascoside	3.40	3.40	95.98	3.52
11	Angoroside C	2.13	2.00	96.20	3.60
12	Harpagoside	0.96	2.65	93.50	2.09

**Table 4:** Stability of the 12 investigated compounds.

No.	Compound	2 h	4 h	8 h	12 h	24 h
1	Fumaric acid	94.36%	92.12%	91.12%	91.11%	89.56%
2	Caffeic acid	95.12%	94.16%	93.45%	92.42%	90.12%
3	Catalpol	93.26%	92.15%	91.89%	91.62%	91.02%
4	Aucubin	95.62%	94.26%	93.45%	92.47%	92.41%
5	*p*-Coumaric acid	95.19%	95.13%	94.16%	93.28%	92.52%
6	Ferulic acid	95.47%	94.13%	93.56%	92.45%	91.44%
7	Harpagide	94.82%	93.46%	93.48%	92.89%	90.46%
8	Cinnamic acid	94.56%	93.74%	92.46%	91.85%	90.76%
9	*p*-Methoxycinnamic acid	95.05%	94.85%	94.01%	93.43%	92.55%
10	Verbascoside	95.22%	94.56%	94.11%	93.84%	91.09%
11	Angoroside C	95.62%	94.51%	93.56%	93.01%	91.95%
12	Harpagoside	94.85%	94.02%	93.45%	91.96%	90.41%

**Table 5:** Contents (mg/g) of the 12 bioactive constituents in the tested samples (mean, *n* = 3).

Samples No.	S1	S2	S3	S4	S5	S6	S7	S8	S9	S10	S11	S12
**1**	4.0392	7.2058	4.0743	6.9791	6.2846	5.1340	5.0065	4.2892	3.4245	3.2126	2.8710	6.2400
**2**	0.0384	0.0426	0.0504	0.0476	0.0954	0.0454	0.0696	0.0497	0.0417	0.0474	0.0902	0.0554
**3**	0.3237	1.2525	0.3429	1.2819	0.5380	0.4739	0.6797	0.3993	0.1718	0.1406	0.1808	0.9006
**4**	2.1630	5.5627	2.2488	5.4172	3.8223	3.8136	6.1443	4.1513	1.7512	1.9700	2.6251	4.8096
**5**	0.1200	0.0656	0.0852	0.0518	0.1436	0.1440	0.0952	0.1392	0.0793	0.1035	0.1756	0.0941
**6**	0.1697	0.1011	0.1237	0.1007	0.3092	0.1655	0.0774	0.0864	0.1150	0.1739	0.2235	0.0578
**7**	4.7448	5.7406	4.9089	5.7782	4.8306	5.4402	6.2461	5.8549	5.6238	5.4837	5.5435	5.4678
**8**	0.9588	0.3916	0.8645	0.4033	1.6973	1.2838	1.7912	1.3546	1.7434	1.8369	1.2993	0.5550
**9**	0.0607	0.0303	0.0469	0.0323	0.1314	0.0714	0.1046	0.0819	0.0923	0.0928	0.0831	0.0506
**10**	0.8826	4.9296	1.7116	6.0078	8.8068	2.7054	6.0725	3.0923	0.8555	0.8918	1.1488	5.4924
**11**	2.6142	2.8577	2.5600	2.9020	3.7264	2.9661	3.5884	3.0929	2.8317	2.7346	2.6779	3.1500
**12**	1.3524	1.5232	1.4077	1.5331	1.4426	1.7549	1.9271	1.4715	1.6348	1.7457	1.5483	1.6680
Total	17.4675	29.7034	18.4250	30.5349	31.8280	23.9981	31.8026	24.0633	18.3651	18.4334	18.4670	28.5413

**Table 6:** Quality sequencing of the samples.

Items	S1	S2	S3	S4	S5	S6	S7	S8	S9	S10	S11	S12
Relative grey correlative degree	0.5069	0.4501	0.5435	0.4531	0.5628	0.5035	0.4849	0.5023	0.4691	0.4688	0.5044	0.5222
Quality-ranking	4	12	2	11	1	6	8	7	9	10	5	3

**Table 7:** Summary of the tested samples of Scrophulariae Radix.

NO.	Medicinal Materials Form	Drying Mode	Drying Temperature/°C	‘Sweating’ Days/day
S1	Complete taproot	Oven drying	55	3
S2	Taproot sections	Oven drying	55	3
S3	Complete taproot	Calorifier drying	55	3
S4	Taproot sections	Calorifier drying	55	3
S5	Complete taproot	Oven drying	35	3
S6	Complete taproot	Oven drying	45	3
S7	Complete taproot	Oven drying	65	3
S8	Complete taproot	Oven drying	55	2
S9	Complete taproot	Oven drying	55	4
S10	Complete taproot	Oven drying	55	5
S11	Complete taproot	Oven drying	55	6
S12	Complete taproot	Sun drying	25	3
